# Impact of ADHD as a Risk and a Treatment Factor in Intimate Partner Violence

**DOI:** 10.1192/j.eurpsy.2022.74

**Published:** 2022-09-01

**Authors:** N. Buitelaar

**Affiliations:** De Forensische Zorgspecialisten, De Waag, Utrecht, Netherlands

**Keywords:** Intimate Partner Violence, adhd, Treatment, Underdiagnosis

## Abstract

Intimate partner violence (IPV) has a very high prevalence (25%) in society and has serious consequences for its victims. As former studies showed minimal effectiveness of therapeutic interventions addressing IPV, the Dutch guideline for Familial/Domestic Violence (NVVP, 2008) recommends to focus more on systemic factors and on individual risk factors of IPV. ADHD is one of these individual risk factors. This presentation focuses on the association between ADHD and IPV, presenting data and clinical examples. ADHD was missed in 56% of a sample of forensic outpatients. Reasons for this issue of underdiagnosis of ADHD in case of aggression and IPV are discussed. Also, data of the ITAP (impact of treatment of ADHD on IPV) study are presented, showing that ADHD in offenders of IPV with ADHD scored higher on minor physical aggression, minor and severe psychological aggression and clinician-rated IPV than offenders without ADHD. Further, after a one year treatment of ADHD and IPV, decrease in IPV was mainly associated with decrease in ADHD symptoms. The importance of screening and treatment of ADHD symptoms in all IPV offenders is discussed to make treatment of IPV more effective.

**Disclosure:**

No significant relationships.

